# Supplementary material for: Peroxidase-Mimicking Ir-Te Nanorods for Photoconversion-Combined Multimodal Cancer Therapy
Source: Nanomaterials (Basel). 2023 May 23;13(11):1706. doi: 10.3390/nano13111706 (PMC10254865; doi:10.3390/nano13111706)
Supplement: Supplementary file 1 [file nanomaterials-13-01706-s001.zip › nanomaterials-2405584-supplementary.pdf]

Supplementary Information for

Peroxidase-Mimicking Ir-Te Nanorods for Photoconversion-Combined Multimodal Cancer Therapy

*Gyeonghye Yim[1]‡, Seounghun Kang[2]‡, Subean Kim[1]‡, and Hongje Jang[1]**

[1]Department of Chemistry, Kwangwoon University, 20 Gwangwoon-ro, Nowon-gu, Seoul 01897, Republic of Korea

[2]Department of Chemistry, Seoul National University, Seoul 08826, Republic of Korea

Supporting data.

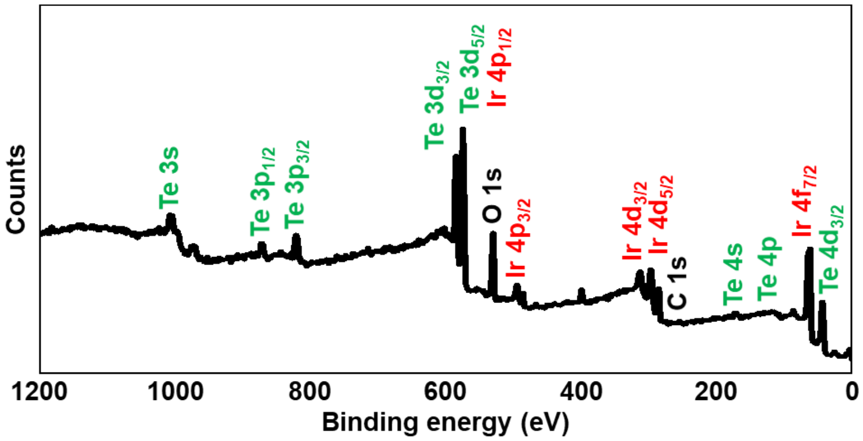

Figure S1. Wide scan XPS survey of IrTeNRs.

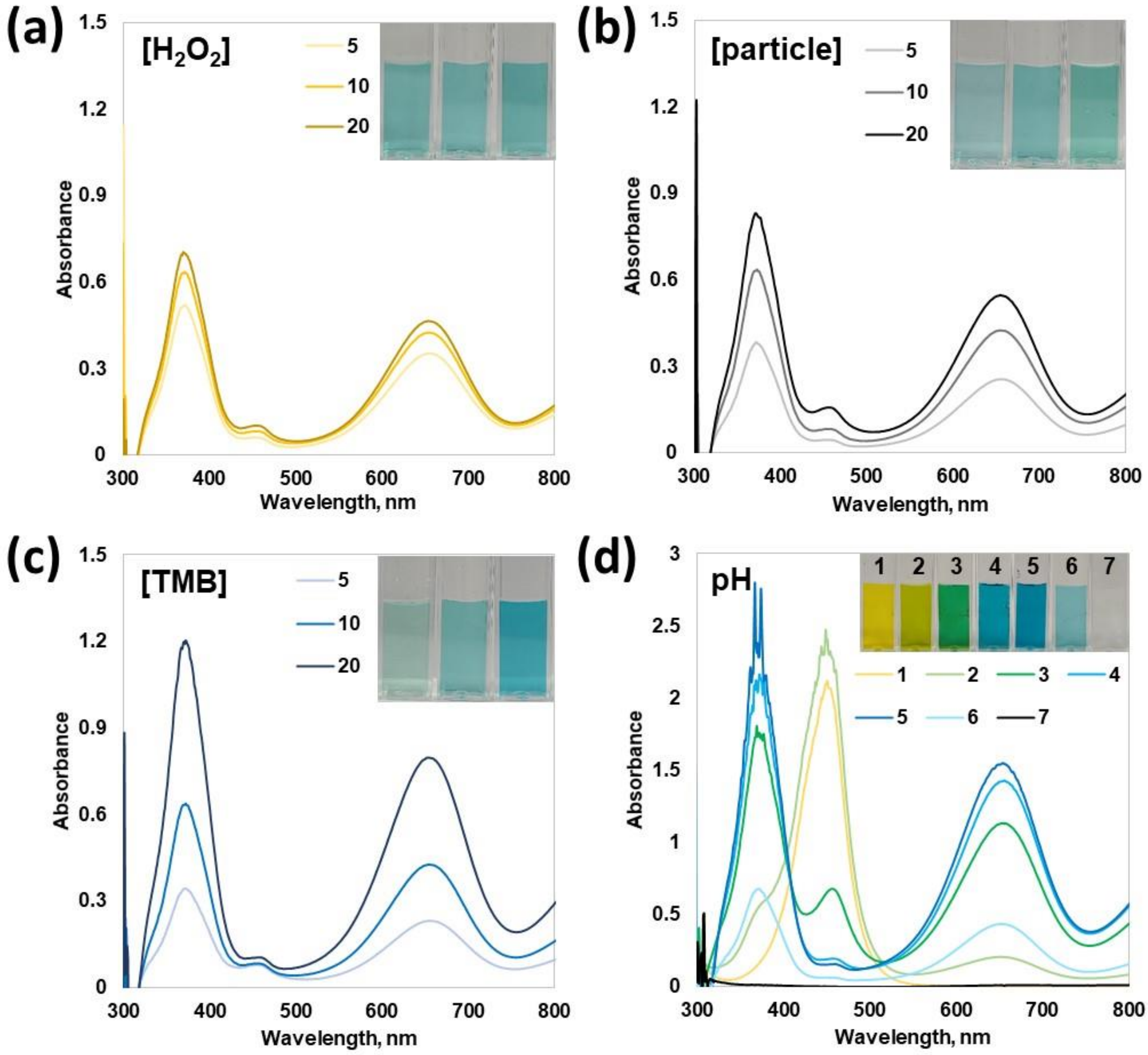

Figure S2. POD-like activities depending on concentration of each factor and pH condition. UV-Vis spectra changes against (a) H_2O_2, (b) IrTeNRs, and (c) TMB concentrations. (d) Optimized pH for POD-like reaction was identified as pH 5.

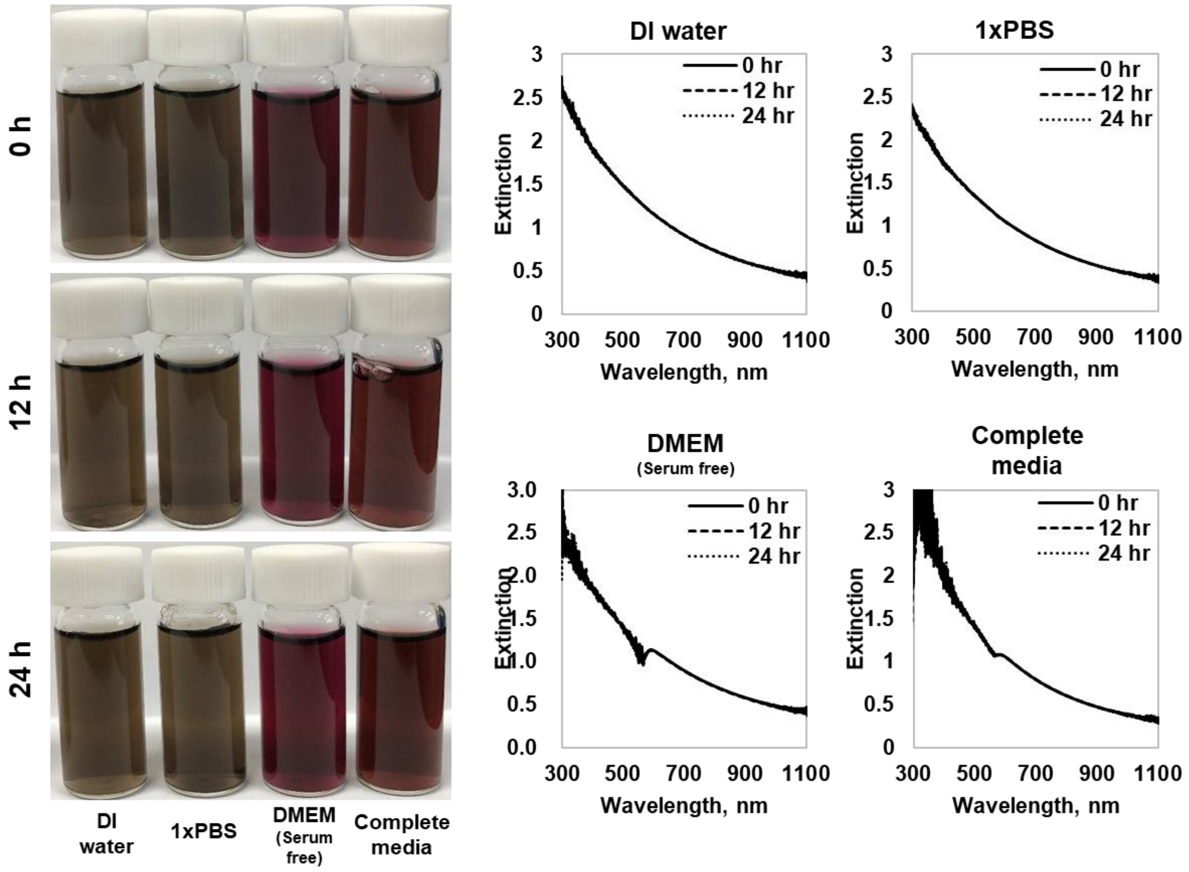

Figure S3. Colloidal stability of IrTeNRs was confirmed by digital photo image (left) and UV-Vis spectra (right) for 24 h against various physiological buffered solutions.

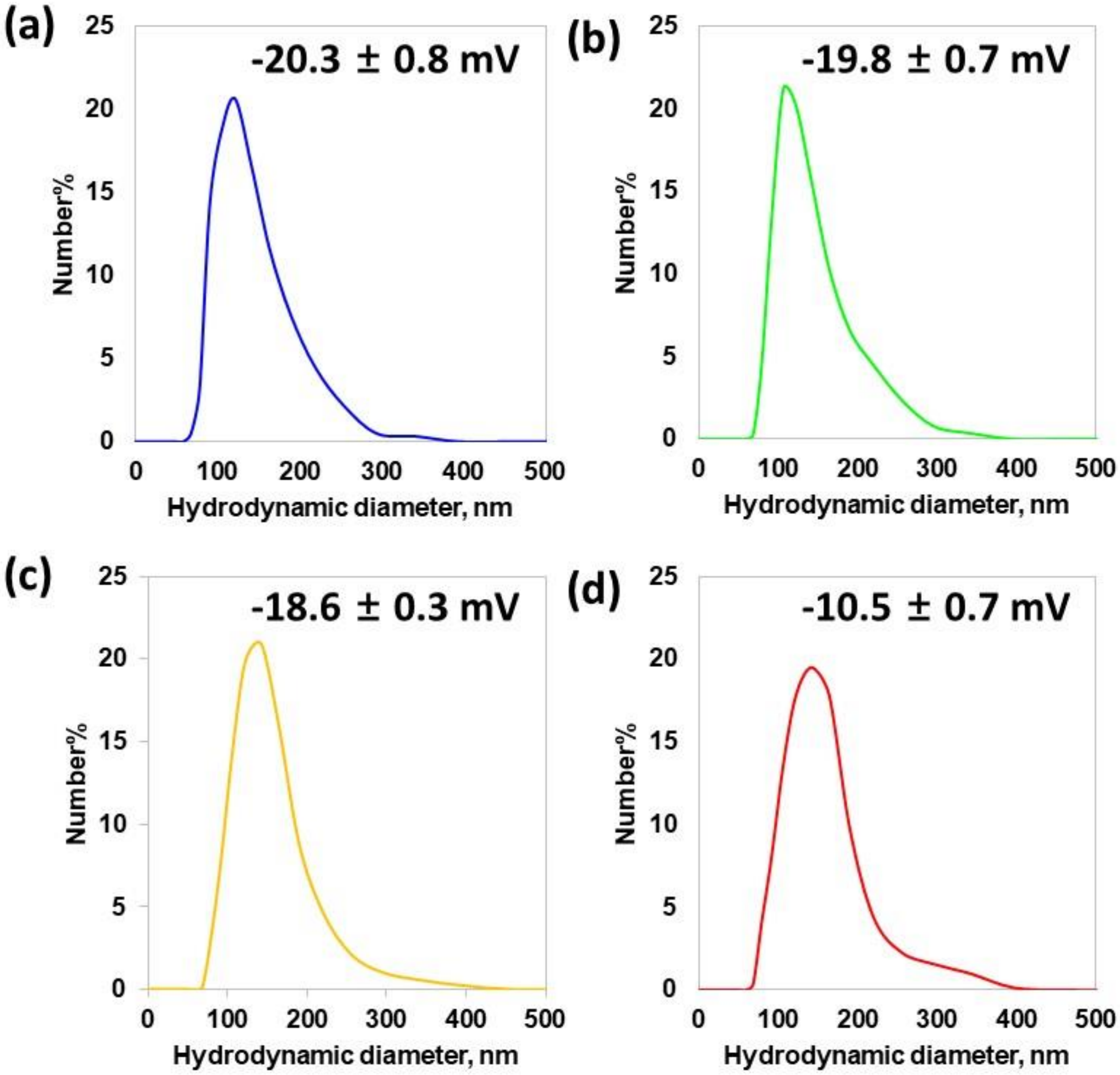

Figure S4. DLS and zeta potential of IrTeNRs which dispersed in (a) DI water, (b) 1xPBS, (c) DMEM, and (d) complete cell culture media.

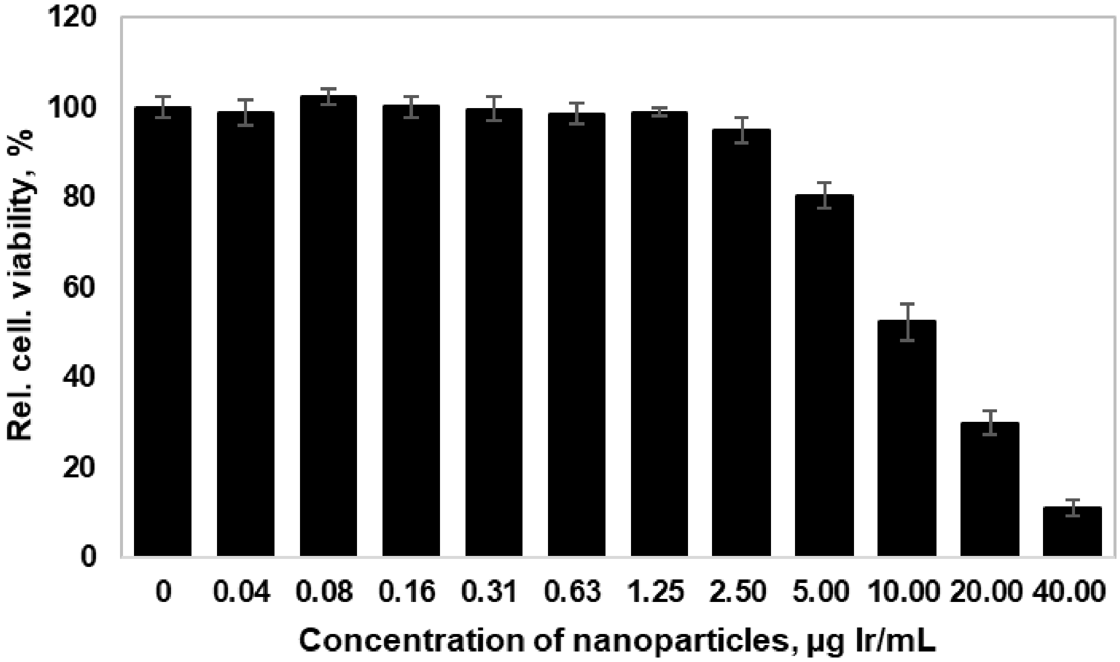

Figure S5. Cytotoxicity of IrTeNRs against HeLa cells.

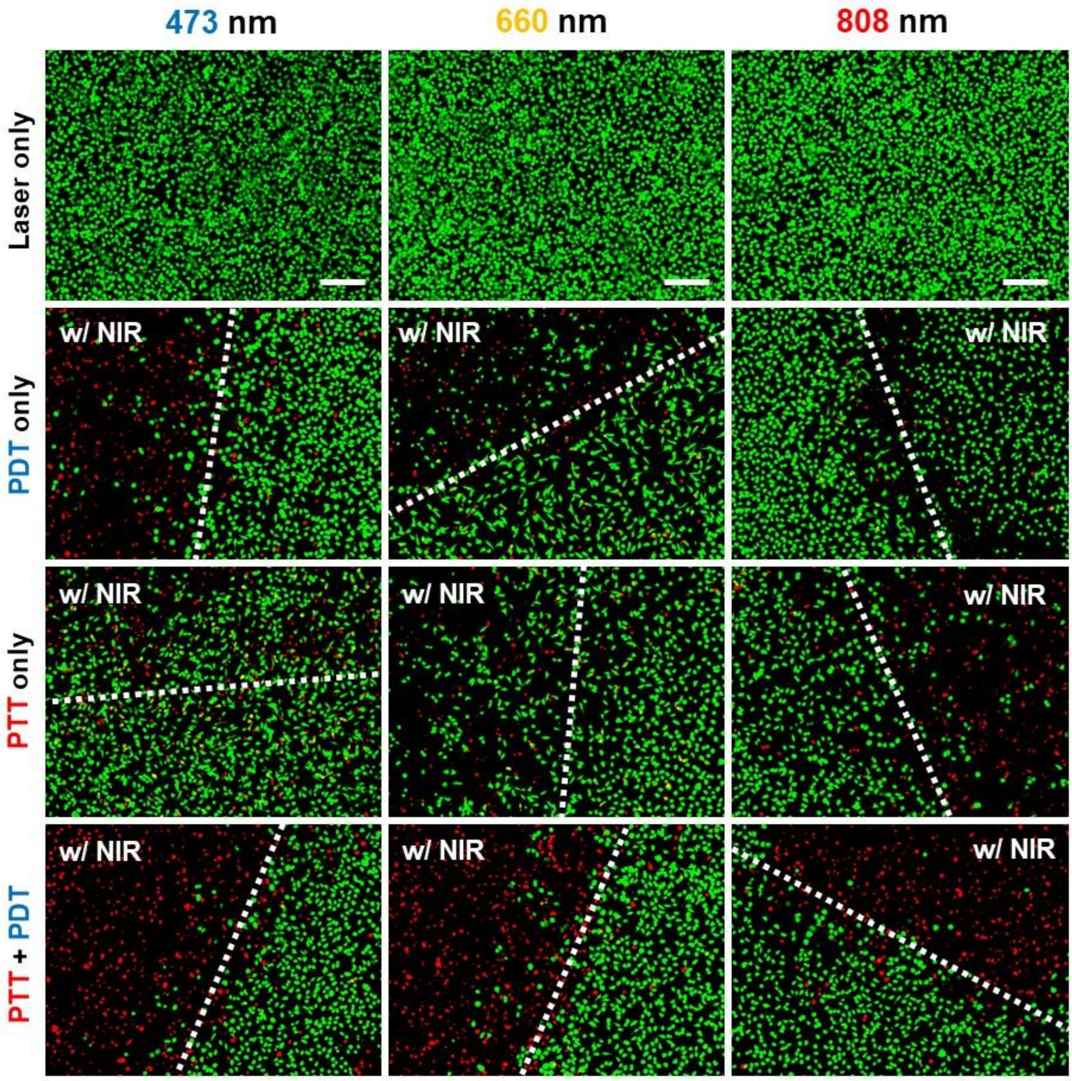

Figure S6. PTT and/or PDT treatment for HeLa cells *in vitro* for IrTeNRs. The scale bar is 250 μm.

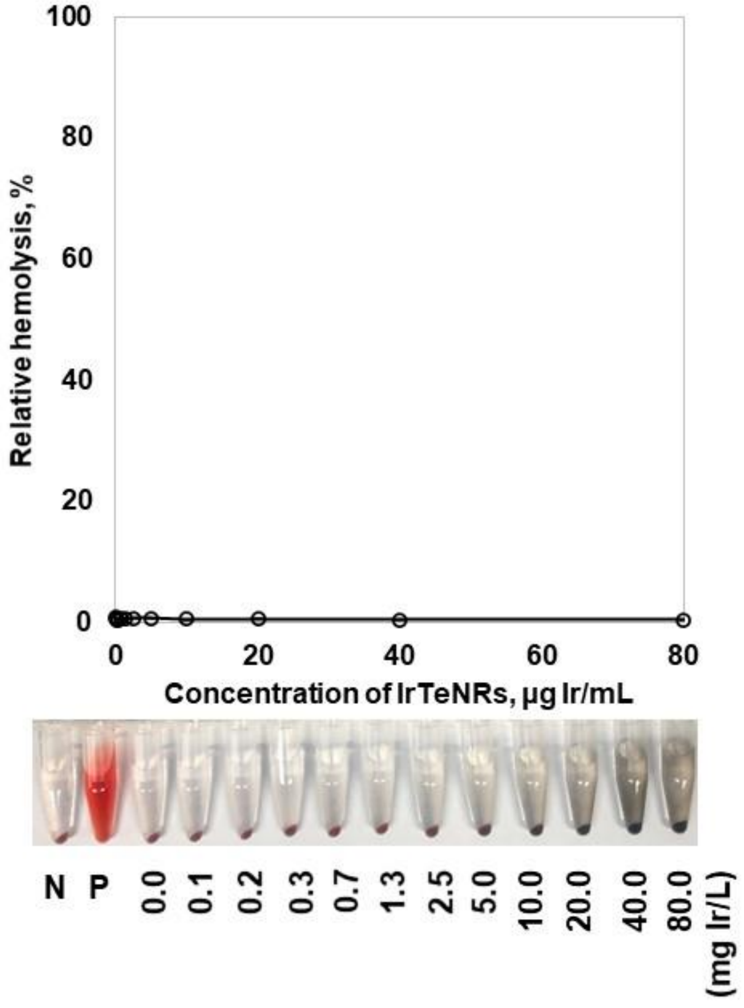

Figure S7. Hemolysis test of IrTeNRs, normalized by the concentration of Ir content.

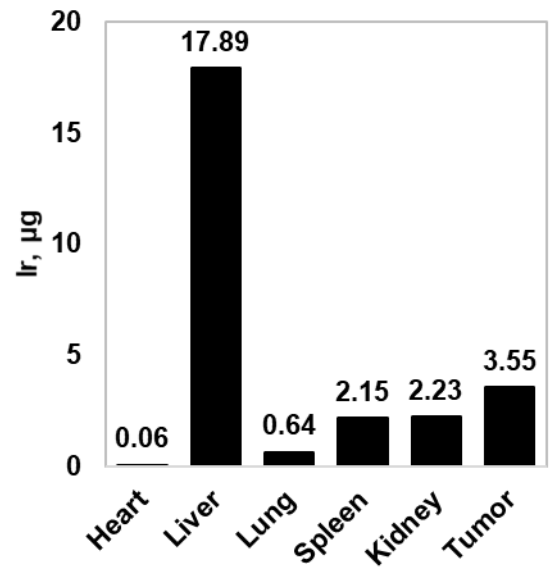

Figure S8. Mouse organ ICP-MS data for biodistribution identification.

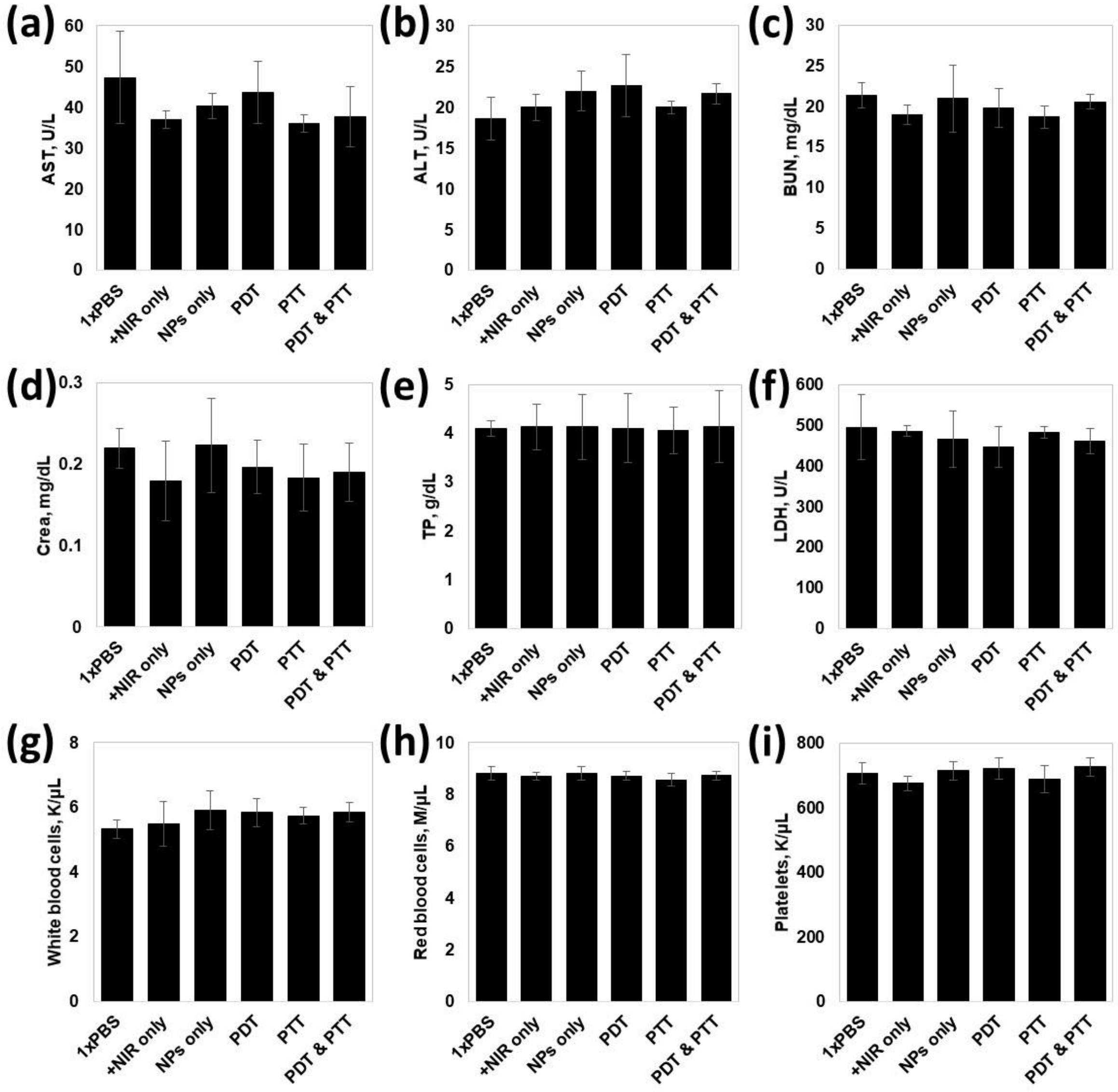

Figure S9. Toxicological profile of IrTeNRs *in vivo*. (a) Aspartate aminotransferase (AST) and (b) alanine aminotransferase (ALT), (c) blood urea nitrogen (BUN) and (d) serum creatine (Crea) showed no liver toxicity and kidney toxicity, respectively. (e) Chronic toxicity indicator total protein (TP) and (f) cell damage biomarker lactate dehydrogenase (LDH) also supported non-toxicological aspect of IrTeNRs against mice model. Hymocytometer result for (g) white blood cells, (h) red blood cells, and (i) platelet counts.

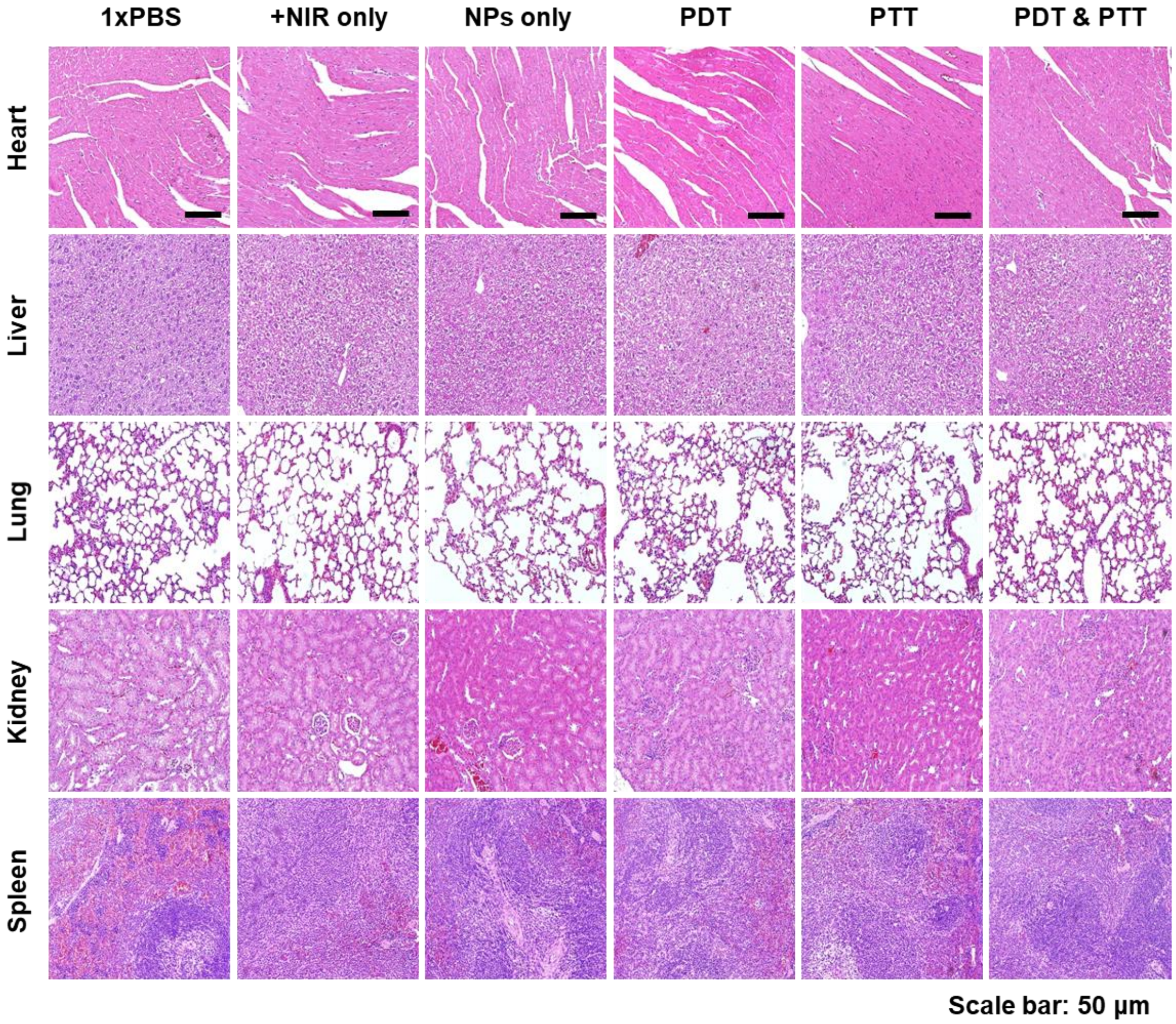

Figure S10. Pathological organ toxicity observations for five major organs against various treatment conditions. The scale bars are 50 μm.

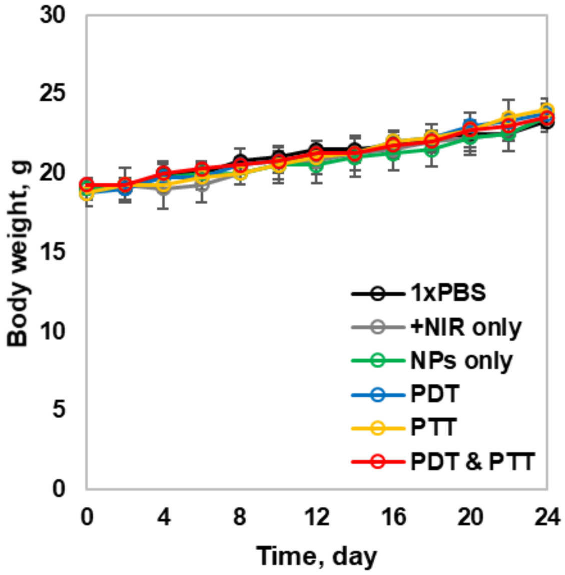

Figure S11. The body weight of mice.
